# Supplementary material for: KRAS, NRAS, BRAF, HER2 and MSI Status in a Large Consecutive Series of Colorectal Carcinomas
Source: Int J Mol Sci. 2023 Mar 2;24(5):4868. doi: 10.3390/ijms24054868 (PMC10003572; doi:10.3390/ijms24054868)
Supplement: Supplementary file 1 [file ijms-24-04868-s001.zip › ijms-2214519-supplementary.pdf]

Table S1. Spectrum and frequency of KRAS, NRAS, and BRAF mutations identified in 8355 CRC cases

**KRAS mutation**	Number of cases	% of cases	Overall frequency	**NRAS mutation**	Number of cases	% of cases	Overall frequency	**BRAF mutation**	Number of cases	% of cases	Overall frequency
Total	4137	100%	49,52%	Total	389	100%	4,66%	Total	556	100%	6,65%
G12D	1193	28.84%	14,28%	Q61K	95	24.42%	1,14%	V600E	509	91.55%	6,09%
G12V	874	21.13%	10,46%	G12D	67	17.22%	0,80%	D594G	24	4.32%	0,29%
G13D	727	17.57%	8,70%	Q61R	59	15.17%	0,71%	D594N	7	1.26%	0,08%
G12C	276	6.67%	3,30%	Q61L	38	9.77%	0,45%	G596R	3	0.54%	0,04%
A146T	235	5.68%	2,81%	G13R	21	5.40%	0,25%	F595L	2	0.36%	0,02%
G12A	206	4.98%	2,47%	Q61H	19	4.88%	0,23%	K601N	2	0.36%	0,02%
G12S	200	4.83%	2,39%	G12V	18	4.63%	0,22%	L597R	2	0.36%	0,02%
Q61H	108	2.61%	1,29%	G13D	18	4.63%	0,22%	D594A	1	0.18%	0,01%
A146V	51	1.23%	0,61%	G12C	14	3.60%	0,17%	D594E	1	0.18%	0,01%
G12R	43	1.04%	0,51%	G12S	12	3.08%	0,14%	K601E	1	0.18%	0,01%
Q61L	35	0.85%	0,42%	G12A	7	1.80%	0,08%	S602F	1	0.18%	0,01%
G13C	24	0.58%	0,29%	G13V	6	1.54%	0,07%	T599dup	1	0.18%	0,01%
Q61R	24	0.58%	0,29%	G13C	2	0.51%	0,02%	T599I	1	0.18%	0,01%
A59T	21	0.51%	0,25%	G60E	2	0.51%	0,02%	V600K	1	0.18%	0,01%
Q61K	21	0.51%	0,25%	A11T	1	0.26%	0,01%				
G13R	10	0.24%	0,12%	A18T	1	0.26%	0,01%				
A146P	6	0.15%	0,07%	A59T	1	0.26%	0,01%				
A59E	6	0.15%	0,07%	A66V	1	0.26%	0,01%				
A59G	6	0.15%	0,07%	E62K	1	0.26%	0,01%				
V14I	6	0.15%	0,07%	E63D	1	0.26%	0,01%				
A18D	5	0.12%	0,06%	G12D+G12V	1	0.26%	0,01%				
G12F	5	0.12%	0,06%	G12R	1	0.26%	0,01%				
L19F	5	0.12%	0,06%	G15E	1	0.26%	0,01%				
Q22K	5	0.12%	0,06%	Q61E	1	0.26%	0,01%				
G60D	4	0.10%	0,05%	Y64C	1	0.26%	0,01%				
Q61P	4	0.10%	0,05%								
G12D+G12V	2	0.05%	0,02%								
G12D+G13D	2	0.05%	0,02%								
G13dup	2	0.05%	0,02%								

G13S	2	0.05%	0,02%
A59del	1	0.02%	0,01%
A59T+A146T	1	0.02%	0,01%
A66X	1	0.02%	0,01%
E62K	1	0.02%	0,01%
E63del	1	0.02%	0,01%
G10_A11dup	1	0.02%	0,01%
G10dup	1	0.02%	0,01%
G10R	1	0.02%	0,01%
G10V	1	0.02%	0,01%
G12D+A146T	1	0.02%	0,01%
G12D+G12C	1	0.02%	0,01%
G12D+V14I	1	0.02%	0,01%
G12L	1	0.02%	0,01%
G12Rfs*22	1	0.02%	0,01%
G12S+G13D	1	0.02%	0,01%
G12S+G13D+Q61H	1	0.02%	0,01%
G12Sfs*22	1	0.02%	0,01%
G12V+G12S	1	0.02%	0,01%
G12V+G13D	1	0.02%	0,01%
G12V+V14I	1	0.02%	0,01%
G13_V14delinsDI	1	0.02%	0,01%
G13D+A59T	1	0.02%	0,01%
G13V	1	0.02%	0,01%
G60_Q61delinsE	1	0.02%	0,01%
K147E	1	0.02%	0,01%
L19_T20delinsFS	1	0.02%	0,01%
Q61D	1	0.02%	0,01%
Q61R+E62G	1	0.02%	0,01%
T58I	1	0.02%	0,01%

Table S2. Associations between the studied genetic alterations, age and gender. Shown are frequencies with standard deviations (SD)

	**KRAS mutation**	**NRAS mutation**	**BRAF mutation**	**MSI**	**HER2 amplification**
**Gender**					
Female	2188/4204 (52.1±0.8%)	168/4204 (4.0±0.3%)	352/4204 (8.4±0.4%)	225/4204 (5.4±0.3%)	51/4006 (1.3±0.2%)
Male	1949/4151 (47.0±0.8%)	221/4151 (5.3±0.3%)	204/4151 (4.9±0.3%)	207/4151 (5.0±0.3%)	48/4002 (1.2±0.2%)
Difference between groups, p	**< 0.0001**	**0.004**	**< 0.0001**	0.459	0.842
**Age groups**					
17-30	19/45 (42.2±7.4%)	2/45 (4.4±3.1%)	1/45 (2.2±2.2%)	10/45 (22.2±6.2%)	2/43 (4.7±3.2%)
31-40	123/288 (42.7±2.9%)	11/288 (3.8±1.1%)	18/288 (6.3±1.4%)	38/288 (13.2±2.0%)	6/271 (2.2±0.9%)
41-50	369/796 (46.4±1.8%)	36/796 (4.5±0.7%)	39/796 (4.9±0.8%)	48/796 (6.0±0.8%)	10/766 (1.3±0.4%)
51-60	909/1925 (47.2±1.1%)	79/1925 (4.1±0.5%)	139/1925 (7.2±0.6%)	90/1925 (4.7±0.5%)	20/1834 (1.1±0.2%)
61-70	1764/3458 (51.0±0.9%)	172/3458 (5.0±0.4%)	217/3458 (6.3±0.4%)	141/3458 (4.1±0.3%)	40/3322 (1.2±0.2%)
71-80	835/1613 (51.8±1.2%)	76/1613 (4.7±0.5%)	122/1613 (7.6±0.7%)	87/1613 (5.4±0.6%)	19/1552 (1.2±0.3%)
> 81	112/217 (51.6±3.4%)	13/217 (6.0±1.6%)	19/217 (8.8±1.9%%)	16/217 (7.4±1.8%)	0/207 (0%)
Difference between groups, p	**0.003**	0.747	0.091	**< 0.0001**	0.156

Table S3. Distribution of mutation frequencies in different Russian Federal Districts (FD). Shown are frequencies with standard deviations (SD)

	**KRAS mutation**	**NRAS mutation**	**BRAF mutation**	**MSI**	**HER2 amplification**	**Any of the studied alterations**
Far Eastern FD	300/654 (45.9±1.9%)	29/654 (4.4±0.8%)	55/654 (8.4±1.1%)	36/654 (5.5±0.9%)	7/649 (1.1±0.4%)	391/654 (59.8±1.9%)
Volga FD	1065/2171 (49.1±1.1%)	101/2171 (4.7±0.5%)	139/2171 (6.4±0.5%)	122/2171 (5.6±0.5%)	26/2139 (1.2±0.2%)	1321/2171 (60.8±1.0%)
Northwestern FD	973/1872 (52.0±1.2%)	84/1872 (4.5±0.5%)	157/1872 (8.4±0.6%)	89/1872 (4.8±0.5%)	24/1636 (1.5±0.3%)	1229/1872 (65.7±1.1%)
North Caucasus FD	455/901 (50.5±1.7%)	38/901 (4.2±0.7%)	44/901 (4.9±0.7%)	52/901 (5.8±0.8%)	8/879 (0.9±0.3%)	544/901 (60.4±1.6%)
Siberian FD	78/172 (45.4±3.8%)	12/172 (7.0±1.9%)	11/172 (6.4±1.9%)	10/172 (5.8±1.8%)	0/170 (0%)	101/172 (58.7±3.8%)
Ural FD	159/336 (47.3±2.7%)	13/336 (3.9±1.1%)	27/336 (8.0±1.5%)	20/336 (6.0±1.3%)	4/328 (1.2±0.6%)	202/336 (60.1±2.7%)
Central FD	701/1424 (49.2±1.3%)	73/1424 (5.1±0.6%)	84/1424 (5.9±0.6%)	64/1424 (4.5±0.5%)	21/1402 (1.5±0.3%)	873/1424 (61.3±1.3%)
Southern FD	406/825 (49.2±1.7%)	39/825 (4.7±0.7%)	39/825 (4.7±0.7%)	39/825 (4.7±0.7%)	9/805 (1.1±0.4%)	492/825 (59.6±1.7%)
**Total**	4137/8355 (49.5±0.5%)	389/8355 (4.7±0.2%)	556/8355 (6.7±0.3%)	432/8355 (5.2±0.2%)	99/8008 (1.2±0.1%)	5153/8355 (61.7±0.5%)
Difference between groups, p	0.163	0.796	**0.001**	0.710	0.706	0.016

Table S4. Frequency of combined genetic alterations in 8355 CRC cases

**KRAS+KRAS**	**KRAS+NRAS**	**KRAS+BRAF**	**KRAS+HER2**	**NRAS+NRAS**	**NRAS+HER2**	**MSI+KRAS**	**MSI+NRAS**	**MSI+BRAF**
13 (0.16%)	8 (0.10%)	4 (0.05%)	12 (0.15%)	1 (0.01%)	4 (0.05%)	138 (1.70%)	9 (0.23%)	117 (1.40%)

Table S5. Comparison of KRAS, NRAS, BRAF mutation frequencies in the current study and in the cBioPortal database

	Current study	cBioPortal*	Difference, p
KRAS	4137/8355 (49.5%)	1704/3815 (44.7%)	<0.0001
NRAS	389/8355 (4.7%)	188/3815 (4.9%)	0.543
BRAF	556/8355 (6.7%)	405/3815 (10.6%)	<0.0001
Co-occurrence of mutations			
KRAS+NRAS	8/8355 (0.10%)	29/3815 (0.76%)	
KRAS+BRAF	4/8355 (0.05%)	45/3815 (1.18%)	
NRAS+BRAF	0/8355 (0.0%)	6/3815 (0.16%)	
Total	12/8355 (0.14%)	80/3815 (2.10%)	<0.0001

*Available at: https://www.cbioportal.org [Accessed: 16 February 2023]

Table S6. Association between KRAS, NRAS, BRAS mutations and gender in the current study and in the cBioPortal database

	Current study	Difference, p	cBioPortal*	Difference, p
	**KRAS**			
Male	1949/4151 (47.0%)	<0.0001	794/1869 (42.5%)	0.022
Female	2188/4204 (52.1%)		831/1796 (46.3%)	
	**NRAS**			
Male	221/4151 (5.3%)	0.004	100/1869 (5.4%)	0.325
Female	168/4204 (4.0%)		83/1796 (4.6%)	
	**BRAF**			
Male	204/4151 (4.9%)	<0.0001	167/1869 (8.9%)	0.0002
Female	352/4204 (8.4%)		229/1796 (12.8%)	

*Available at: https://www.cbioportal.org [Accessed: 16 February 2023]

Table S7. Methods for detection of KRAS, NRAS, BRAF mutations, MSI, and HER2 amplification and overexpression

Gene / Mutation	Primers	PCR composition	PCR conditions / Device
**High resolution melting (HRM) analysis[a]**			
KRAS: fragment of exon 2, which contains codons 12 and 13	Forward: AATGACTGAATATAAACTTGTGG<br>Reverse: [Biotin]CAAGATTTACCTCTATTGTTGG<br>Pyrosequencing primer: AATGACTGAATATAAACTTGTGG	1 µl template, 0.15 u uracil-DNA glycosylase, 0.75 u hot-start polymerase, GeneAmp™ 10X PCR Buffer I (Applied Biosystems), 3.5 mM $MgCl_2$, 1x EvaGreen, 200 µM dNTPs, 0.3 µM primers in a total volume of 20 µl	DNA processing with uracil-DNA glycosylase: 15 min., 37°C; activation of Taq polymerase: 10 min., 95°C; then 50 cycles (denaturation: 15 s, 95°C; annealing: 30 s, 60°C; synthesis: 30 s, 72°C); then high-resolution melting analysis (temperature ramping from 65 to 95°C, rising by 0.07°C/1 s and fluorescence acquisition setting recommended by the manufacturer). Device: LightCycler® 96 Instrument (Roche)
KRAS: fragment of exon 3, which contains codons 59 and 61	Forward: [Biotin]TGTTTCTCCCTTCTCAGGATTC<br>Reverse: GTACTGGTCCCTCATTGCAC<br>Pyrosequencing primer: GTCCCTCATTGCACTGT		
KRAS: fragment of exon 4, which contains codon 146	Forward: [Biotin]GTAGACACAAAACAGGCTCAG<br>Reverse: TGTATTTATTTCAGTGTTACTTAC<br>Pyrosequencing primer: TGTTACTTACCTGTCTTG		
NRAS: fragment of exon 2, which contains codons 12 and 13	Forward: TTGCTGGTGTGAAATGACTG<br>Reverse: [Biotin]CACTGGGCCTCACCTCTA<br>Pyrosequencing primer: TACAAACTGGTGGTGGT		
NRAS: fragment of exon 3, which contains codon 61	Forward: TATAGATGGTGAAACCTGTTTG<br>Reverse: [Biotin]ATGGCAAATACACAGAGGAAG<br>Pyrosequencing primer: GTTTGTTGGACATACTGG		
BRAF: fragment of exon 15, which contains codon 600	Forward: CCTTTACTTACTACACCTCAG<br>Reverse: [Biotin]CACAAAATGGATCCAGACAACT<br>Pyrosequencing primer: GACCTCACAGTAAAAATAG		
**Allele-specific real-time PCR**			

KRAS: codons 12 and 13	Wild-type: CTTGTGGTAGTTGGAGCTGG<br>Mutant:<br>KRAS p.G12D: CTTGTGGTAGTTGGAGCTGA<br>KRAS p.G12V: CTTGTGGTAGTTGGAGCTGT<br>KRAS p.G13D: GGTAGTTGGAGCTGGTGA<br>KRAS p.G12C: ACTTGTGGTAGTTGGAGCTT<br>KRAS p.G12A: CTTGTGGTAGTTGGAGCTGC<br>KRAS p.G12S: CTTGTGGTAGTTGGAGCTA<br>KRAS p.G12R: CTTGTGGTAGTTGGAGCTC<br>KRAS p.G13C: TTGTGGTAGTTGGAGCTGGTT<br>Common: CAAGATTTACCTCTATTGTTGG	1 µl template, 0.75 u hot-start polymerase, GeneAmp™ 10X PCR Buffer I (Applied Biosystems), 2.0 mM $MgCl_2$, 0.2x SYBR Green I, 200 µM dNTPs, 0.3 µM primers in a total volume of 17 µl	Activation of Taq polymerase: 10 min., 95°C; then 50 cycles (denaturation: 15 s, 95°C; annealing: 30 s, 62°C; synthesis: 30 s, 72°C).<br>Device: CFX-96 (Bio-Rad)
KRAS: codons 59 and 61	Wild-type: CTGTACTCCTCTTGACCTGC<br>Mutant:<br>KRAS p.A59G: CACTGTACTCCTCTTGACCTC<br>KRAS p.A59T: CTGTACTCCTCTTGACCTGT<br>KRAS p.Q61H_C: TCATTGCACTGTACTCCTCG<br>KRAS p.Q61H_T: CTCATTGCACTGTACTCCTCA<br>KRAS p.Q61L: TCATTGCACTGTACTCCTCTA<br>KRAS p.Q61R: TCATTGCACTGTACTCCTCTC<br>KRAS p.Q61K: TCATTGCACTGTACTCCTCTTT<br>Common: GACTGTGTTTCTCCCTTCTCA	1 µl template, 0.75 u hot-start polymerase, GeneAmp™ 10X PCR Buffer I (Applied Biosystems), 2.0 mM $MgCl_2$, 0.2x SYBR Green I, 200 µM dNTPs, 0.3 µM primers in a total volume of 20 µl	Activation of Taq polymerase: 10 min., 95°C; then 50 cycles (denaturation: 15 s, 95^0C; annealing: 30 s, 62°C; synthesis: 30 s, 72 °C).<br>Device: CFX-96 (Bio-Rad)
KRAS: codon 146	Wild-type: ACTTACCTGTCTTGTCTTTGC<br>Mutant:<br>KRAS p.A146T: ACTTACCTGTCTTGTCTTTGT<br>KRAS p.A146P: ACTTACCTGTCTTGTCTTTGG<br>KRAS p.A146V: GTTACTTACCTGTCTTGTCTTTA<br>Common: AGATGTACCTATGGTCCTAGTA	1 µl template, 0.75 u hot-start polymerase, GeneAmp™ 10X PCR Buffer I (Applied Biosystems), 2.5 mM $MgCl_2$, 0.2x SYBR Green I, 200 µM dNTPs, 0.3 µM primers in a total volume of 20 µl	Activation of Taq polymerase: 10 min., 95°C; then 50 cycles (denaturation: 15 s, 95°C; annealing: 30 s, 62°C; synthesis: 30 s, 72 °C)<br>Device: CFX-96 (Bio-Rad)
NRAS: codons 12 and 13	Wild-type: GCGCTTTTCCCAACACCAC<br>Mutant:<br>NRAS p.G12D: GCGCTTTTCCCAACACCAT<br>NRAS p.G12V: GCGCTTTTCCCAACACCAA<br>NRAS p.G12C: CGCTTTTCCCAACACCACA<br>NRAS p.G12A: GCGCTTTTCCCAACACCAG<br>NRAS p.G12S: CGCTTTTCCCAACACCACT<br>NRAS p.G13D: CAGTGCGCTTTTCCCAACAT<br>NRAS p.G13R: AGTGCGCTTTTCCCAACACG<br>Common: GCCAATTAACCCTGATTACTG	1 µl template, 0.75 u hot-start polymerase, GeneAmp™ 10X PCR Buffer I (Applied Biosystems), 2.0 mM $MgCl_2$, 0.2x SYBR Green I, 200 µM dNTPs, 0.3 µM primers in a total volume of 20 µl	Activation of Taq polymerase: 10 min., 95°C; then 50 cycles (denaturation: 15 s, 95°C; annealing: 30 s, 62°C; synthesis: 30 s, 72°C).<br>Device: CFX-96 (Bio-Rad)

NRAS: codon 61	Wild-type: CATACTGGATACAGCTGGACA<br>Mutant:<br>NRAS p.Q61R: CATACTGGATACAGCTGGACG<br>NRAS p.Q61K: ACATACTGGATACAGCTGGAA<br>NRAS p.Q61L: CATACTGGATACAGCTGGACT<br>NRAS p.Q61H_T: ATACTGGATACAGCTGGACAT<br>NRAS p.Q61H_C: TACTGGATACAGCTGGACAC<br>Common: TACACAGAGGAAGCCTTCGC	1 µl template, 0.75 u hot-start polymerase, GeneAmp™ 10X PCR Buffer I (Applied Biosystems), 2.5 mM $MgCl_2$, 0.2x SYBR Green I, 200 µM dNTPs, 0.3 µM primers in a total volume of 20 µl	Activation of Taq polymerase: 10 min., 95°C; then 50 cycles (denaturation: 15 s, 95°C; annealing: 30 s, 62°C; synthesis: 30 s, 72°C)<br>Device: CFX-96 (Bio-Rad)
BRAF: codon 600	Wild-type: GGTGATTTTGGTCTAGCTACAGT<br>Mutant:<br>BRAF p.V600E: GGTGATTTTGGTCTAGCTACAGA<br>BRAF p.V600K: GGTGATTTTGGTCTAGCTACAA<br>Common: ATAGCCTCAATTCTTACCATCC	1 µl template, 0.75 u hot-start polymerase, GeneAmp™ 10X PCR Buffer I (Applied Biosystems), 2.5 mM $MgCl_2$, 0.2x SYBR Green I, 200 µM dNTPs, 0.3 µM primers in a total volume of 20 µl	Activation of Taq polymerase: 10 min., 95°C; then 50 cycles (denaturation: 15 s, 95°C; annealing: 30 s, 62°C; synthesis: 30 s, 72°C)<br>Device: CFX-96 (Bio-Rad)
**Digital droplet PCR**			
KRAS: codons 12 and 13	Forward: AAATGACTGAATATAAACTTGT<br>Reverse: ATTAGCTGTATCGTCAAGG<br>Probes:<br>Wild-type: [FAM]CCTACGCCACCAGCTC[BHQ1]<br>Mutant:<br>KRAS p.G12D: [R6G]CCTACGCCATCAGCTC[BHQ1]<br>KRAS p.G12V: [R6G]CCTACGCCAACAGCTC[BHQ1]<br>KRAS p.G13D: [R6G]CCTACGTCACCAGCTC[BHQ1]<br>KRAS p.G12C: [R6G]CCTACGCCACAAGCTC[BHQ1]<br>KRAS p.G12A: [R6G]CCTACGCCAGCAGCTC[BHQ1]<br>KRAS p.G12S: [R6G]CCTACGCCACTAGCTC[BHQ1]<br>KRAS p.G12R: [R6G]CCTACGCCACGAGCTC[BHQ1]<br>KRAS p.G13C: [R6G]CCTACGCAACCAGCTC[BHQ1]	2 µl template, 4 µl ddPCR Supermix for Probes (no dUTP) (Bio-Rad), 0.5 µM primers and probes in a total volume of 20 µl	Activation of Taq polymerase: 10 min., 95°C; then 50 cycles (denaturation: 30 s, 94°C; annealing/synthesis: 60 s, 56°C), then heating: 10 min., 98°C.<br>Device: T100 Thermal Cycler (Bio-Rad); QX200 Droplet Digital PCR System (Bio-Rad)

KRAS: codons 59 and 61	Forward: GACACAGCAGGTCAAGAG<br>Reverse: TCCTCATGTACTGGTCCC<br>Probes:<br>Wild-type: [FAM]ACTCCTCTTGACCTGCTGT[BHQ1]<br>Mutant:<br>KRAS p.A59G: [JOE]ACTCCTCTTGACCTCCTGT[BHQ1]<br>KRAS p.A59T: [JOE]ACTCCTCTTGACCTGTTGT[BHQ1]<br>KRAS p.Q61H_C: [R6G]ACTCCTCGTGACCTGCTGT[BHQ1]<br>KRAS p.Q61H_T: [R6G]ACTCCTCATGACCTGCTGT[BHQ1]<br>KRAS p.Q61L: [R6G]ACTCCTCTAGACCTGCTGT[BHQ1]<br>KRAS p.Q61R: [R6G]ACTCCTCTCGACCTGCTGT[BHQ1]<br>KRAS p.Q61K: [R6G]ACTCCTCTTTACCTGCTGT[BHQ1]
KRAS: codon 146	Forward: CTTAGCAAGAAGTTATGGAAT<br>Reverse: GTATTTATTTCAGTGTTACTTAC<br>Probes:<br>Wild-type: [FAM]AACATCAGCAAAGACAAGACA[BHQ1]<br>Mutant:<br>KRAS p.A146T: [R6G]AACATCAACAAAGACAAGACA[BHQ1]<br>KRAS p.A146P: [R6G]AACATCACCAAAGACAAGACA[BHQ1]<br>KRAS p.A146V: [R6G]AACATCAGTAAAGACAAGACA[BHQ1]
NRAS: codons 12 and 13	Forward: CTTGCTGGTGTGAAATGAC<br>Reverse: ATTGTCAGTGCGCTTTTCC<br>Probes:<br>Wild-type: [FAM]ACACCACCTGCTCCAACCAC[BHQ1]<br>Mutant:<br>NRAS p.G12D: [R6G]ACACCATCTGCTCCAACCAC[BHQ1]<br>NRAS p.G12V: [JOE]ACACCAACTGCTCCAACCAC[BHQ1]<br>NRAS p.G12C: [JOE]ACACCACATGCTCCAACCAC[BHQ1]<br>NRAS p.G12A: [JOE]ACACCAGCTGCTCCAACCAC[BHQ1]<br>NRAS p.G12S: [JOE]ACACCACTTGCTCCAACCAC[BHQ1]<br>NRAS p.G13D: [JOE]ACATCACCTGCTCCAACCAC[BHQ1]<br>NRAS p.G13R: [JOE]ACACGACCTGCTCCAACCAC[BHQ1]

NRAS: codon 61	Forward: ACCTGTTTGTTGGACATACT<br>Reverse: ATTGGTCTCTCATGGCACT<br>Probes:<br>Wild-type: [FAM]ACAGCTGGACAAGAAGAGTACAGT[BHQ1]<br>Mutant:<br>NRAS p.Q61R: [HEX]ACAGCTGGACGAGAAGAGTACAGT[BHQ1]<br>NRAS p.Q61K: [HEX]ACAGCTGGAAAAGAAGAGTACAGT[BHQ1]<br>NRAS p.Q61L: [JOE]ACAGCTGGACTAGAAGAGTACAGT[BHQ1]<br>NRAS p.Q61H_T: [JOE]ACAGCTGGACATGAAGAGTACAGT[BHQ1]<br>NRAS p.Q61H_C: [JOE]ACAGCTGGACACGAAGAGTACAGT[BHQ1]		
BRAF: codon 600	Forward: GAAGACCTCACAGTAAAAATAG<br>Reverse: TCAAACTGATGGGACCCACT<br>Probes:<br>Wild-type: [FAM]TCTAGCTACAGTGAAATCTCGATGG[BHQ1]<br>Mutant:<br>BRAF p.V600E: [HEX]TCTAGCTACAGAGAAATCTCGATGG[BHQ1]<br>BRAF p.V600K: [HEX]TCTAGCTACAAAGAAATCTCGATGG[BHQ1]		
**Quantitative real-time PCR**			
HER2	Amplification:<br>HER2:<br>Forward: CCAAACTAGCCCTCAATCC<br>Reverse: ATCTTCTGCTGCCGTCGCTT<br>Probe: [FAM]CATTCTGCTGGTCGTGGTCTTGG[BHQ1]<br>TMEM (gene-referee):<br>Forward: TGTGTGCTATTAACTGTGGAAA<br>Reverse: GTAACGCCACAGGACAATC<br>Probe: [R6G]CTCCTTCTCCTAGTTTGATGCCA[BHQ1]<br><br>Expression:<br>HER2:<br>Forward: GATCTTTGGGAGCCTGGCAT<br>Reverse: AAACACTTGGAGCTGCTCTG<br>Probe: [FAM]CTGCCGGAGAGCTTTGATGGGGAC[BHQ1]<br>SDHA (gene-referee):<br>Forward: CCACTCGCTATTGCACACC<br>Reverse: ATCCAAGGCAAAATACTCCAC<br>Probe: [JOE]CTGGTATCATATCGCAGAGACC[BHQ1]	1 µl template, 0.75 u hot-start polymerase, GeneAmp™ 10X PCR Buffer I (Applied Biosystems), 2.5 mM MgCl2, 200 µM dNTPs, 0.3 µM primers and probes in a total volume of 20 µl	Activation of Taq polymerase: 10 min., 95°C; then 45 cycles (denaturation: 15 s, 95°C; annealing/synthesis: 60 s, 60°C)<br>Device: CFX-96 (Bio-Rad)
**Fragment analysis of mononucleotide microsatellite markers**			

MSI[b]	BAT26 (182bp):<br>Forward: CTGCGGTAATCAAGTTTTTAG<br>Reverse: AACCATTCAACATTTTTAACCC<br>BAT25 (153bp):<br>Forward: TACCAGGTGGCAAAGGGCA<br>Reverse: TCTGCATTTTAACTATGGCTC<br>NR21 (109bp):<br>Forward: GAGTCGCTGGCACAGTTCTA<br>Reverse: CTGGTCACTCGCGTTTACAA<br>NR22 (143bp):<br>Forward: GAGGCTTGTCAAGGACATAA<br>Reverse: AATTCTGATGCCATCCAGTT<br>NR24 (128bp):<br>Forward: GCTGAATTTTACCTCCTGAC<br>Reverse: ATTGTGCCATTGCATTCCAA	1 µl template, 0.75 u hot-start polymerase, GeneAmp™ 10X PCR Buffer I (Applied Biosystems), 2.5 mM $MgCl_2$, 200 µM dNTPs, 0.3 µM primers, in a total volume of 20 µl	Activation of Taq polymerase: 10 min., 95°C; then 38 cycles (denaturation: 20 s, 95°C; annealing: 30 s, 58°C; synthesis: 30 s, 72°C)<br>Device: CFX-96 (Bio-Rad) or LightCycler® 96 Instrument (Roche)<br>Fragment analysis, device: GenomeLab GeXP Genetic Analysis System (Beckman Coulter)

[a]High-resolution melting was used as the initial screening method to detect mutations in KRAS exons 2, 3, 4, NRAS exons 2, 3, and BRAF exon 15. When the melting curve/peak deformation clearly indicated the presence of heteroduplexes, but no common variants were detected by allele-specific PCR, PCR product was directly subjected to the DNA pyrosequencing.

[b]6324 samples were tested using one mononucleotide repeat marker BAT-26. 2031 samples were tested using five mononucleotide repeat markers (BAT-25, BAT-26, NR-21, NR-22, and NR-24). When two or more markers were unstable, the sample was interpreted as MSI, all other samples were classified as microsatellite stable (MSS).
